# Association between urinary albumin-to-creatinine ratio within the normal range and continuous glucose monitoring-derived metrics in children and adolescents with type 1 diabetes

**DOI:** 10.1186/s13098-025-01749-x

**Published:** 2025-05-26

**Authors:** László Barkai, Olivér Rácz, György Eigner, Levente Kovács

**Affiliations:** 1 Department of Paediatrics and Adolescent Medicine, Faculty of Medicine, Pavol Jozef Šafárik University, Kosice, Slovakia; 2 Institute of Pathophysiology, Faculty of Medicine, Pavol Jozef Šafárik University, Kosice, Slovakia; 3 Biomatics and Applied Artificial Intelligence Institute, John von Neumann Faculty of Informatics, Obuda University, Budapest, Hungary; 4 Physiological Controls Regulation Research Center, University Research and Innovation Center, Obuda University, Budapest, Hungary

**Keywords:** Type 1 diabetes, Children, Adolescents, Continuous glucose monitoring, Albumin-to-creatinin ratio

## Abstract

**Aims:**

Albuminuria within the normal range may predict an increased risk of subsequent nephropathy in type 1 diabetes (T1D). The role of sustained hyperglycaemia in the development of nephropathy is well-known. The relationship between albuminuria within the normal range and parameters of continuous glucose monitoring (CGM) in childhood has not yet been investigated. The aim of the present study was to analyze this relationship in young T1D patients.

**Methods:**

A total of 54 normoalbuminuric, normotensive, real time CGM user pubertal children and adolescents with T1D were recruited for this study. Patients with medium to high normal (1.0-2.9 mg/mmol; *n* = 18) and those with low normal (< 1.0 mg/mmol; *n* = 36) urinary albumin-to-creatinin ratio (UACR) were compared regarding CGM metrics data. Relationships of UACR with clinical variables and CGM-derived metrics were analysed by multiple logistic regression.

**Results:**

Time in range (TIR) was lower in medium to high normal UACR patients than in low normal UACR patients (mean ± SD: 58.2 ± 8.4% vs. 64.5 ± 10.1%, *p* = 0.0199). Patients with medium to high normal UACR had a higher coefficient of variation for mean glucose (CV) than those with low normal UACR (42.4 ± 6.0% vs. 38.0 ± 6.1%, *p* = 0.0163). UACR was related to TIR (*r*=-0.55, *p* = 0.02), to CV (*r*=-0.51, *p* = 0.04) and to mean glucose (MG) (*r*=-0.48, *p* = 0.05). TIR, CV and puberty proved to be independently predictive for medium to high normal UACR [adjusted RR (95% CI): 0.70 (0.58–0.92), *p* = 0.0231; 1.28 (1.02–1.67), *p* = 0.0222; 1.19 (1.10–1.36), *p* = 0.0321, respectively].

**Conclusion:**

The duration of the blood glucose level within the target range and the extent of its fluctuation may contribute to the early increase in albumin excretion within the normal range, which may play a role in the development of later complications of childhood T1D.

## Introduction

Chronic complications of diabetes can only rarely be diagnosed in the form of clinical symptoms in children and adolescents. However, early functional abnormalities in the affected organs and tissues can be present already at this young age, after a few years of diabetes [1]. Over the past decades, data have emerged showing that the incidence of certain late complications in childhood diabetes is decreasing [2, 3]. Given that diabetes manifests at an increasingly earlier age, the chance of developing certain complications is significant throughout life. It is also well known in clinical practice that the treatment of children and adolescents faces particular difficulties due to physiological reasons, therefore metabolic control at this age is generally worse than in adults and this circumstance favors the early progression of chronic complications [4].

In clinical terms, the earliest manifestation of diabetes kidney disease is the appearance of albuminuria, which predicts the proteinuria that will appear later in the course of the disease [5, 6]. In addition, previous studies show that increases in albumin excretion within the normal range can predict cardiovascular disease in both healthy and diabetes individuals [7].

The role of overall hyperglycemia, as measured by glycated hemoglobin (HbA1c), in the pathogenesis of diabetes kidney disease is well established. Experimental studies have demonstrated that glucose fluctuations are more deleterious to human endothelial cells than sustained hyperglycemia [8]. Previous studies in adults using continuous glucose monitoring (CGM) suggested a role for glycemic variability (GV) in diabetes nephropathy [9].

To our knowledge, till now the relationship between standardised CGM metrics for clinical care and albumin excretion within the normal range in children and adolescents has not yet been investigated. Therefore, in this study, the association between CGM-derived glucometrics and the urinary albumin-to-creatinin ratio (UACR) in the normal range was investigated in a group of children and adolescents with T1D.

## Methods

### Study design and participants

This was a cross sectional study. A total of 54 children and youths with T1D were recruited for this study (Újbuda Szent Kristóf Diabetes Center, Budapest, Hungary). Patients fulfilled the following inclusion criteria: (1) age ≥ 10 years, (2) duration of diabetes ≥ 1 year, (3) normoalbuminuria, (4) no clinical evidence of diabetes complications, (5) absence of acute infecious disease or chronic condition other than diabetes, (6) normal blood pressure for age, sex and height by clinical measurements.

Patients were excluded if they had type 2 or monogenic diabetes, obesity (BMI ≥ 95th percentile or ≥ 30 kg/m2 for patient ≥ 18 years of age), chronic inflammatory diseases, acute infection, or a history of malignancy. None of the subjects had any disorder apart from diabetes; and none of them showed any clinical symptoms indicating neuropathy or angiopathy. Patients with microalbuminuria, subclinical retinopathy (on fundus photography or fluorescein angiography), cardiovascular autonomic (assessed by cardiovascular tests) or peripheral nerve dysfunction (assessed by quantitative sensory nerve testing) were excluded from the study. Patients had not been treated with any medicine (except for insulin) during the preceding 2 weeks. Individuals who were engaged in competition sport or participated in exercise training programmes were excluded. All patients were in stable metabolic condition with no ketonuria and no ketoacidosis during the previous one year. Normotension was defined as both systolic and diastolic BP of < 90th percentile for the age, sex and height of the subject.

The age of the patients was between 10 and 22 years (16.0 ± 3.0 years) and they had diabetes for at least one year. Duration of diabetes was between 1 and 14 years (8.0 ± 2.3 years). All subjects entered puberty (Tanner stage ≥ 2). All of the patients were normoalbuminuric (UACR < 3.0 mg/mmol) and were treated with insulin using pen or pump. They used real time CGM routinely and needed to wear their device > 70% of the time and upload data on a monthly basis. All parents and children were encouraged by the diabetes care team to achieve optimal metabolic control. All were encouraged to measure four blood glucoses daily. All were trained in carbohydrate counting and diabetes self-care. Patients underwent formal physical examination during clinic visit.

Two groups were selected according to the UACR: patients with medium to high normal (1.0-2.9 mg/mmol) and patients with low normal (< 1.0 mg/mmol) result [10]. The two study groups were matched for age, sex, diabetes duration and most recent HbA1c. The study was approved by the Central Ethics Committee, Budapest, Hungary (BM/19256-2/2024).

### Assessments

After exclusion of proteinuria due to urinary tract infection, albuminuria was assessed as the UACR in a first morning urine collection. Urinary albumin was measured by turbidimetry and creatinine by enzymatic method with spectrophotometry. Electronic medical records were reviewed for the most recent HbA1c within 1 month of enrollment. Records were also reviewed for a recent serum creatinine (Cr) value within one year of enrollment to estimate the renal function. However, not all patients had creatinine level within the previous one year, therefore this parameter was not included in the later analysis.

Blood pressure was measured using a sphygmomanometer with the subject in the seated position using appropriate sized cuff and after 5-min rest. Height was measured using a stadiometer and weight using an electronic scale. BMI, height, and blood pressure were expressed as standard deviation scores (SDS). SDSs were calculated according to the formula (X_i_ - M_x_)/S_x_, where X_i_ is the actual measurement, M_x_ is the mean value for that age/height and sex, and S_x_ is the standard deviation corresponding to that age/height and sex. Pubertal development was assessed according to Tanner criteria (breast and pubic hair stages for girls; genitalia and pubic hair stages for boys) rating in five stages (Tl-5).

All patients were experienced RT-CGM user (≥ 3 months) thus adequate glucose data were available for evaluation (70% use of CGM over 14 days). The glucose profiles were analyzed according to the following parameters: time in range (TIR; percentage of time glucose is within range 3.9–10.0 mmol/L), time above range (TAR; percentage of time glucose is above range 3.9–10.0 mmol/L), time below range (TBR; percentage of time glucose is below range 3.9–10.0 mmol/L), mean glucose (MG), coefficient of variation for mean glucose (CV) and glucose management index (GMI) [11].

Subjects with type 1 diabetes were using real-time CGM or iCGM in conjunction with intensive insulin therapy as Multiple Daily Insulin injection therapy (MDI) or Continuous Subcutaneous Insulin Infusion regimen (CSII) /Advanced Hybrid Closed Loop systems (AHCLs) for at least 6 months prior the study with a sensor use > 70%. Insulin administration was applied with pen for MDI and pump (Tandem t: slim X2 with Control IQ technology or Medtronic 640G, 780G) for CSII/AHCLs. Medtronic Enlite, Guardian, Freestyle Libre2 and Dexcom G6 sensors were used and Carelink (Medtronic Diabetes, Inc., Northridge, CA), Libreview (version 3.10; Abbott Diabetes Care, Ltd., Witney, U.K.) and Clarity (Dexcom International Switzerland, Horw, Switzerland) were used as web-based platforms. Two patients in the medium to high normal UACR group and 3 patients in the low normal UACR group used AHCLs.

### Statistical analysis

Clinical characteristics and results of study groups are expressed as mean ± SD. Distribution was tested for each variable by the Kolmogorov-Smirnov test. UACR was skewedly distributed and logarithmically transformed before analysis. Data were analysed using SPSS 26.0 statistical software (IBM Corp., Armonk, NY, USA). Significance level was set at *P* ≤ 0.05. For comparisons between groups, t-test or Mann-Whitney test were used for continuous variables and Fisher’s exact test was performed for categorical relationship within study groups. The correlations between UACR and clinical and RT-CGM-derived parameters were assessed by Spearman correlation. Multiple logistic regression analysis was applied to assess relationships of UACR with clinical variables and RT-CGM-derived metrics.

## Results

Clinical characteristics according to urinary albumin-to-creatinin ratio of youths with type 1 diabetes are shown on Table 1. The two groups were comparable regarding age, sex, diabetes duration and most recent glycemic control. Four patients in the medium to high normal UACR group and 17 patients in the low normal UACR group were in the early puberty (Tanner stages 2–3), respectively. Six patients in the medium to high normal UACR group and 10 patients in the low normal UACR group were treated with pump therapy, respectively. Pubertal staging, treatment modalities were comparable in the two groups. BMI, height, daily insulin dose, systolic or diastolic blood pressure did not differ significantly between the medium to high normal and low normal UACR groups.

**Table 1:** Clinical characteristics according to urinary albumin-to-creatinin ratio of youths with type 1 diabetes (mean ± SD)

	Urinary albumin-to-creatinin ratio		*p*
	medium to high normal (1.0-2.9 mg/mmol)	low normal (< 1.0 mg/mmol)	
Number of patients (males)	18 (8)	36 (16)	1.0000
Age, years	16.1 ± 3.2	16.0 ± 2.9	0.9118
Tanner stages: 2–3/4–5	4/14	17/19	0.1376
Diabetes duration, years	8.2 ± 2.3	8.0 ± 2.4	0.7683
HbA1c % mmol/mol	7.5 ± 0.9 58 ± 6.96	7.3 ± 1.0 56 ± 7.67	0.4631
Pump therapy (n)	6	10	0.7560
BMI (SDS)	0.3 ± 0.3	0.2 ± 0.2	0.2127
Height (SDS)	0.2 ± 0.3	0.3 ± 0.3	0.2562
Insulin dose (U/kg/day)	0.8 ± 0.2	0.7 ± 0.3	0.1522
SBP (SDS)	0.1 ± 0.3	0.1 ± 0.4	0.9442
DBP (SDS)	0.2 ± 0.6	0.3 ± 0.3	0.8682

BMI: body mass index

SBP: systolic blood pressure

DBP: diastolic blood pressure

SDS: standard deviation score

Data are expressed as number or mean ± standard deviation

Time in range was lower in medium to high normal UACR patients than in low normal UACR patients (58.2 ± 8.4% vs. 64.5 ± 10.1%, *p* = 0.0199). Patients with medium to high normal UACR had a higher coefficient of variation for mean glucose than those with low normal UACR (42.4 ± 6.0% vs. 38.0 ± 6.1%, *p* = 0.0163). Mean glucose and time above range tended to be higher in the medium to high normal UACR group than in the low normal UACR group, but did not reach statistical significance. Time below range and glucose management index did not differ in the study groups (Table 2.). UACR of the patients was significantly related to time in range (*r*=-0.55, *p* = 0.02), to coefficient of variation for mean glucose (*r*=-0.51, *p* = 0.04) and to mean glucose values (*r*=-0.48, *p* = 0.05). No association was found between UACR and clinical parameters and other RT-CGM-derived parameters as time above range, time below range, and glucose management index.

**Table 2:** Continuous glucose monitoring-derived metrics of youths with type 1 diabetes (mean ± SD)

	Urinary albumin-to-creatinin ratio		*p*
	medium to high normal (1.0-2.9 mg/mmol) *n* = 18	low normal (< 1.0 mg/mmol) *n* = 36	
TIR (%)	58.2 ± 8.4	64.5 ± 10.1	0.0199
TAR (%)	35.8 ± 11.1	30.2 ± 10.4	0.0838
TBR (%)	5.3 ± 2.1	5.1 ± 2.0	0.7397
CV (%)	42.4 ± 6.0	38.0 ± 6.1	0.0163
MG (mmol/l)	8.9 ± 1.3	8.1 ± 1.6	0.0557
GMI (%)	7.6 ± 1.3	7.2 ± 1.4	0.3058

TIR: time in range

TAR: time above range

TBR: time below range

CV: coefficient of variation for mean glucose

MG: mean glucose

GMI: glucose management index

Data are expressed as mean ± standard deviation

To assess which factors were associated with the medium to high normal UACR, multiple logistic regression analysis was performed on the combined cohort of diabetes patients using the presence of medium to high normal UACR as the dependent variable. The initial model included sex, puberty, diabetes duration, most recent HbA1c, BMI, height, daily insulin dose, systolic and diastolic blood pressure and CGM-derived metrics investigated as independent variables. Puberty, TIR and CV remained in a model that was highly predictive of medium to high normal UACR. In this multivariate analysis, TIR, CV and puberty represented an independent risk of the medium to high normal UACR as compared with the low normal UACR (Table 3).

**Table 3:** Models associated with medium to high normal albumin-to-creatinin ratio in youths with type 1 diabetes

	Explanatory variable	Univariate		Multivariate	
		RR (95% CI)	*P*	adjusted RR (95% CI)	*P*
Medium to high normal UACR	TIR	0.69 (0.54–0.89)	0.0185	0.70 (0.58–0.92)	0.0231
	CV	1.35 (1.03–1.60)	0.0423	1.28 (1.02–1.67)	0.0222
	Late puberty	1.18 (1.09–1.38)	0.0450	1.19 (1.10–1.36)	0.0321

UACR: urinary albumin-to-creatinin ratio

TIR: time in range

CV: coefficient of variation for mean glucose

Late puberty: Tanner stage 4–5

RR: relative risk

CI: confidence interval

## Discussion

Clinical renal complications (overt nephropathy, end-stage renal disease) are very rare in children and adolescents with type 1 diabetes, but early, preclinical functional and structural abnormalities (hyperfiltration, silent or incipient nephropathy) may develop relatively early after the diagnosis of diabetes and may progress rapidly during puberty. This supports the importance of research aimed at identifying early signs of renal complications in young patients and predicting later progression.

In this study, the association between CGM-derived glucometrics and the UACR in the normal range was investigated in a group of children and adolescents with T1D. Higher UACR was associated with lower TIR and higher CV. TIR, CV and puberty were independently predictive for medium to high normal UACR. These results showed that the duration of the blood glucose level within the target range and the extent of its fluctuation may contribute to the early increase in albumin excretion within the normal range.

Currently, the appearance of albuminuria is considered the first clinical sign of diabetes nephropathy, which is also considered a risk factor for further progression [5, 6]. According to some data, an increase in albumin excretion already within the normal range can predict the appearance of cardiovascular diseases in both diabetes and healthy individuals [7]. Among children and adolescents with T1D, the early increase in albumin excretion in the years following the diagnosis of diabetes may predict the subsequent increase in albumin and protein excretion [12]. Taking all these into account, an increase in early albumin excretion within the normal range in youths with T1D can be a useful marker of the development of later kidney complications. However, further prospective studies are needed to evaluate this relationship.

The pathological role of persistent hyperglycemia in the development of diabetes kidney disease is well known. Nevertheless, according to experimental data, glycemic fluctuations may be even more harmful to endothelial cells than persistently high blood glucose levels [4]. In clinical practice, self-monitoring of blood glucose is an excellent way to assess metabolic control and predict complications, however, according to the data of the Diabetes Control and Complications Trial, blood glucose fluctuations that can be determined in this way do not play a significant role in the development of complications [13, 14].

With the widespread use of continuous glucose monitoring, new indicators of blood glucose control and glycemic variability have been introduced in clinical practice, which can be considered as early markers and predictors of diabetes complications [11]. Previous data obtained in adults using CGM suggested a role for glycemic variability in diabetes nephropathy [5]. A recent investigation documented association between CGM-derived time in range and urinary albumin-to-creatinin ratio in T1D adult patients with albuminuria [15]. Furthermore, a pilot study investigated urinary biomarkers of early kidney damage (neutrophil gelatinase-associated lipocalin and pentosidine) in children and adolescents with T1D and suggested that glucose variability obtained by CGM may play a role in the process of diabetes nephropathy [16]. Our present study is in line with these findings showing that higher glycemic fluctuation could be an important mediator of the early increase in the albumin excretion. The specific contribution of glucose variability to the disease process in various stages of diabetes nephropathy is yet to be elucidated.

Although glycemic variability may play a role in the development of diabetes kidney complications, clear evidence of this is still lacking and the underlying mechanism by which blood glucose fluctuations contribute to the development of certain complications independently of persistent hyperglycemia is also unknown [17]. The risk of developing diabetes complications can only be partially explained by the degree of long-term hyperglycemia reflected by HbA1c, therefore it seems that the measure of blood glucose variability can provide useful data for further refining the risk [18]. According to some data, short-term blood glucose swings induce oxidative stress, initiate inflammatory processes and result in epigenetic changes, which may play a role in the pathogenesis of chronic complications of diabetes [19]. It seems that persistent hyperglycemia causes increased protein glycation, and short-term blood glucose changes exert their effects through oxidative stress, inflammation, endothelial dysfunction, and altered gene expression [20, 21, 22].

In our study, in addition to glycemic variability indices, puberty was also independently associated with higher UACR within the normal range. Previous studies showed that puberty may speed up the development of albumin excretion rate above the normal range [12, 23]. Our present finding suggests that puberty may be an additional factor in accelerating the development of the earliest stage of nephropathy, when albumin excretion is still in the normal range.

Our study had limitations. First, this study was retrospective and cross-sectional and lacks long-term follow-up. Second, in this study, we analyzed the results of a single UACR determination, as in routine clinical practice, a negative result is not always repeated. Third, although acute infection was excluded, a mild, incipient, asymptomatic infection could not be ruled out. Furthermore, although we did not include participants in an exercise program, mild physical activity could not be excluded and possible changes in diet could not be controlled for. These conditions could also have influenced albumin excretion. Therefore, caution is required when interpreting the results.

Other parameters of renal function were not analyzed in our study primarily because creatinine levels within one year were not available in all cases. Some data suggest that reduced glomerular filtration rate is not uncommon in normoalbuminuric T1D children [24, 25], so further studies are needed to determine how glucose fluctuations affect filtration.

## Conclusions

In summary, this study showed that shorter duration of blood glucose within the target range and the more pronounced fluctuation of glycemia independent of the high long-term average glucose level may contribute to an early increase in albumin excretion within the normal range in children and adolescents with T1D. Higher glucose variability and pubertal development confer an independent risk of early progress of diabetes kidney injury, which may play a role in the development of later diabetes nephropathy.

## Data Availability

No datasets were generated or analysed during the current study.
